# Autophagy and Cancer: Insights into Molecular Mechanisms and Therapeutic Approaches for Chronic Myeloid Leukemia

**DOI:** 10.3390/biom15020215

**Published:** 2025-02-02

**Authors:** Mohd Adnan Kausar, Sadaf Anwar, Yusuf Saleem Khan, Ayman A. Saleh, Mai Ali Abdelfattah Ahmed, Simran Kaur, Naveed Iqbal, Waseem Ahmad Siddiqui, Mohammad Zeeshan Najm

**Affiliations:** 1 Department of Biochemistry, College of Medicine, University of Ha’il, Hail 55476, Saudi Arabia; sa.anwar@uoh.edu.sa; 2 Department of Anatomy, College of Medicine, University of Ha’il, Hail 55476, Saudi Arabia; y.salem@uoh.edu.sa; 3 Department of Pathology, College of Medicine, University of Ha’il, Hail 55476, Saudi Arabia; aym.ali@uoh.edu.sa; 4 Department of Pediatrics, College of Medicine, University of Ha’il, Hail 55476, Saudi Arabia; mai.ahmed@uoh.edu.sa; 5 School of Biosciences, Apeejay Stya University, Sohna, Gurugram 122103, Haryana, India; s.kaur010707@gmail.com; 6 Department of Obstetrics and Gynecology, College of Medicine, University of Ha’il, Ha’il 55476, Saudi Arabia; n.ulhaq@uoh.edu.sa; 7 Interdisciplinary Biotechnology Unit, Aligarh Muslim University, Aligarh 202001, Uttar Pradesh, India; waseem.ibu@amu.ac.in

**Keywords:** autophagy, chronic myeloid leukemia, BCR-ABL, autophagosome, tyrosine kinase inhibitors

## Abstract

Autophagy is a critical cellular process that maintains homeostasis by recycling damaged or aberrant components. This process is orchestrated by a network of proteins that form autophagosomes, which engulf and degrade intracellular material. In cancer, autophagy plays a dual role: it suppresses tumor initiation in the early stages but supports tumor growth and survival in advanced stages. Chronic myeloid leukemia (CML), a hematological malignancy, is characterized by the Philadelphia chromosome, a chromosomal abnormality resulting from a translocation between chromosomes 9 and 22. Autophagy has emerged as a key factor in CML pathogenesis, promoting cancer cell survival and contributing to resistance against tyrosine kinase inhibitors (TKIs), the primary treatment for CML. Targeting autophagic pathways is being actively explored as a therapeutic approach to overcome drug resistance and enhance cancer cell death. Recent research highlights the intricate interplay between autophagy and CML progression, underscoring its role in disease biology and treatment outcomes. This review aims to provide a comprehensive analysis of the molecular and cellular mechanisms underlying CML, with a focus on the therapeutic potential of targeting autophagy.

## 1. Introduction

Autophagy is a cellular mechanism that maintains homeostasis by eliminating invasive microorganisms, damaged organelles, and protein aggregates through lysosomal degradation [1]. This process involves the formation of an autophagosome, a double-membrane vesicle that encapsulates cellular components for degradation. As a lysosome-dependent catabolic process, autophagy is activated in response to cellular stresses such as nutrient starvation, oxygen deprivation, or pathogen invasion. It plays a dual role by recycling cytoplasmic components during stress and operating at baseline levels under normal physiological conditions. The dysregulation or imbalance of autophagy has been associated with the onset of various conditions, including neurodegenerative diseases, metabolic disorders, and cancer [2].

Chronic myeloid leukemia (CML) is a myeloproliferative neoplasm driven by a chromosomal translocation between chromosomes 9 and 22. This genetic alteration produces the Philadelphia chromosome, a defining feature of CML. The BCR-ABL fusion protein is persistently active as a result of this translocation, which promotes unchecked cellular proliferation, suppresses apoptosis, and disrupts normal adhesion mechanisms [3]. Emerging evidence highlights the dual role of autophagy in CML progression and treatment response. Autophagy can enhance CML cell survival by providing essential nutrients and energy under stress conditions. Conversely, in specific contexts, such as during chemotherapy, autophagy may shift its role and contribute to cell death [4].

The inhibition of autophagy has emerged as a potential therapeutic strategy for CML. Preclinical trials have demonstrated that blocking autophagy can enhance the efficacy of chemotherapeutic agents in eradicating CML cells [5]. Findings from recent studies investigating the complex interplay between CML progression and autophagy further support the therapeutic potential of targeting this process [6]. Ongoing research aims to identify optimal strategies for modulating autophagy, with an emphasis on their clinical significance in CML treatment. This review provides a critical analysis of the current knowledge on the role of autophagy in the pathogenesis of CML, further evaluating its potential as a novel therapeutic intervention.

## 2. Insights into the Basics of Autophagy

The term “autophagy” was coined over 40 years ago by Christian de Duve, whose pioneering research revealed the process’s fundamental role in cellular biology. De Duve’s work originated from observations in rats, where administering the pancreatic hormone glucagon caused a significant decrease in the number of mitochondria and other vital cellular components within lysosomes [7]. The term itself is derived from the Greek words auto (“self”) and phagy (“to eat”), collectively describing the self-cannibalizing process through which cells degrade their own components. Autophagy facilitates the breakdown and recycling of cytoplasmic macromolecules, including proteins and lipids, providing energy and essential building blocks for cellular functions. This system is crucial for preserving the integrity of the genome, promoting cell survival, and regulating cellular metabolism [8,9]. Additionally, it serves as a cellular housekeeping mechanism, eliminating unwanted or harmful substances such as misfolded or aggregated proteins, oncogenic materials [10], damaged organelles [11,12], and invading pathogens like viruses. These components are degraded through a lysosome-dependent pathway [13].

Autophagy begins with the formation of an autophagosome, a double-membraned vesicle that encapsulates cellular components earmarked for degradation and recycling. These components include misfolded proteins, damaged organelles, and other cellular waste. The autophagosome subsequently fuses with a lysosome, a membrane-bound organelle containing enzymes that degrade the autophagosome’s contents. This tightly regulated process is activated in response to cellular stresses such as infections, oxidative damage, and nutrient deprivation. Autophagy plays a vital role in maintaining cellular homeostasis, particularly in cancer cells, which frequently encounter nutrient shortages and other stressors. However, dysregulated autophagy has been implicated in the pathogenesis of various diseases, including neurodegenerative disorders, metabolic conditions, and cancer.

Autophagy plays a vital role in targeting and eliminating malignant cells and toxic compounds that can contribute to cancer development. This process is essential for maintaining cellular homeostasis and supporting normal growth and development. However, its paradoxical role in cancer presents significant challenges. While autophagy can suppress tumor initiation, it can also promote metastasis and tumor recurrence. Furthermore, dysregulated autophagy enhances the resistance of cancer stem cells to chemotherapeutic agents and radiation, thereby complicating clinical management [14]. In the context of viral infections, autophagy intersects with various host cellular functions, including metabolism, immune responses, and cellular trafficking, which viruses exploit to ensure their survival and replication [15,16]. As a fundamental mechanism for degrading and recycling cellular components, autophagy is integral to both innate and adaptive immune responses, playing a crucial role in defending against viral pathogens [17,18].

The lysosome facilitates the degradation of cellular components through three primary forms of autophagy: micro-autophagy, macro-autophagy, and chaperone-mediated autophagy. In macro-autophagy, cytoplasmic contents are sequestered within the autophagosome, which subsequently fuses with the lysosome to form an autolysosome, where degradation occurs. In contrast, micro-autophagy involves the direct engulfment of cytoplasmic components by invaginations of the lysosomal membrane. Chaperone-mediated autophagy differs from the other two forms by exclusively targeting specific proteins, wherein the chaperone proteins, such as Hsc70, bind to the targeted proteins and transport them to the lysosomal membrane. Interaction with lysosomal-associated membrane protein 2A induces a conformational change that facilitates the translocation of these proteins into the lysosome, where they are degraded [19].

Autophagy has been extensively studied across various fields, including health, disease, degeneration, and hereditary or lifestyle-related conditions [13,20]. Although much of the research has concentrated on its roles in cancer [10], degenerative diseases [21], and microbial infections [13], the scope of autophagy’s functions is now being explored in a broader range of contexts.

## 3. Understanding the Molecular Machinery of Autophagy

Autophagy is regulated by a well-coordinated series of cellular signaling pathways, with various signals and cellular stresses contributing to the stimulation of this process [22]. The initiation of autophagy begins with autophagosome formation—double-membrane vesicles that interact with lysosomes to degrade unnecessary cellular components. The development and functionality of autophagosomes are significantly influenced by autophagy-related genes (ATGs) [23]. The molecular mechanism of an autophagy comprises four distinct phases: initiation, nucleation, maturation, and fusion, followed by the degradation of cellular constituents.

**Figure 1:**
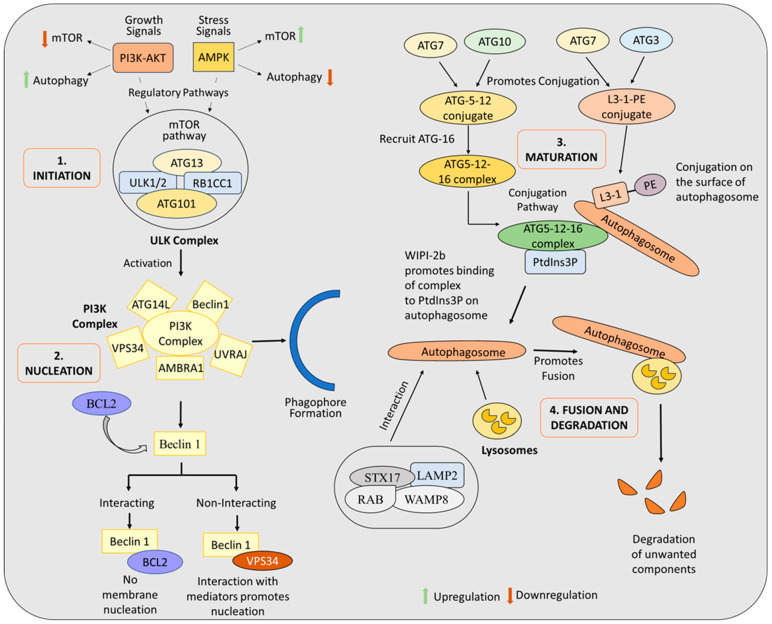
Molecular process of autophagy is delineated into four stages: initiation, nucleation, maturation, and the final steps of fusion and degradation.

The evolutionarily conserved serine/threonine kinase mechanistic target of rapamycin (mTOR) plays a pivotal role in transmitting signals that stimulate autophagy and is essential for initiating the autophagic cascade. mTOR exists in two distinct complexes, mTORC1 and mTORC2, each of which regulates cellular energy homeostasis [24]. Autophagy and mTORC1 exhibit an inverse relationship, where the activation of one suppresses the other. For example, the PI3K/Akt pathway activates mTORC1, which inactivates autophagy by phosphorylating ULK1 (Unc-51-like autophagy-activating kinase) and ATG13, thereby suppressing the autophagic process [25]. Conversely, AMP-activated protein kinase (AMPK) stimulates autophagy by monitoring ATP and cellular energy levels [26]. Reduced ATP levels activate AMPK, which phosphorylates RAPTOR (regulatory-associated protein of mTOR) and TSC2 (TSC complex subunit 2), leading to the inhibition of mTOR and subsequent activation of the ULK1/2 complex [27]. The ULK1/2 complex, comprising ULK1 or ULK2 kinase, ATG13, ATG101, and FIP200 (also known as RB1CC1), works alongside ATG9 to facilitate autophagosome nucleation. The initiation of autophagy begins with the activation of the ULK1/2 complex, which triggers the formation of a double-membrane structure known as the phagophore. This process is mediated by the activation of the class III phosphatidylinositol 3-kinase (PI3K) complex [28] (Figure 1).

The class III PI3K complex is primarily responsible for the nucleation of the autophagic membrane. The phagophore, a double-membrane structure, forms through the regulation of several proteins, including AMBRA1, *Beclin-1*, VPS34, and mATG9 [29,30]. As the phagophore forms, it undergoes conformational changes, elongating and transforming into an isolation membrane, which has a characteristic cup-shaped structure. *Beclin-1* is responsible for regulating membrane nucleation, and its interaction with *Bcl-2* inhibits the process of autophagy. The disruption of the *Bcl-2-Beclin-1* interaction allows *Beclin-1* to associate with VPS34, a lipid kinase, triggering membrane nucleation and facilitating autophagy [31]. In addition to VPS34, *Beclin-1* interacts with various mediators, including the UV radiation resistance-associated gene protein (UVRAG) [32], RUBICON, AMBRA1 [33], and ATG14L [34], to modulate membrane formation. VPS34, a class III phosphatidylinositol 3-kinase enzyme, generates phosphatidylinositol 3-phosphate (PI3P), a critical signaling molecule that drives autophagosome formation [35].

During the maturation phase, the isolation membrane expands and seals, forming a complete autophagosome. The binding of ATGs, facilitated by autophagic proteins within the PI3P-binding domain at the membrane nucleation site, supports the elongation and sealing of the autophagosome membrane. This step involves two critical protein conjugation processes essential for autophagosome formation. In the first step, ATG7 and ATG10 catalyze the conjugation of ATG5 and ATG12 proteins. Following this conjugation, the ATG5-12 assembly recruits ATG16L1, forming the ATG12–ATG5–ATG16L1 complex [36]. This complex binds to PI3P, produced by VPS34, on newly formed autophagosomal membranes through interaction with WIPI-2b [37]. In the second step, ATG7 and *ATG3* conjugate LC3 protein to phosphatidylethanolamine, a lipid molecule [38]. ATG4 cleaves the precursor LC3 protein, exposing glycine residues at its carboxy-terminal, which results in the formation of the soluble LC3-I form. The ATG12–ATG5–ATG16L1 complex is essential for the lipidation of LC3-I to PE on the autophagosomal membrane surface, a process mediated by ATG7 and ATG3 [39]. This lipidation labels the membrane as autophagic by attaching LC3-I, a ubiquitin-like protein, to its surface. LC3-I interacts with cargo receptors, enabling the transport of autophagic cargo to the autophagosome. The lipid-modified version of LC3, referred to as LC3-II, facilitates autophagic degradation by interacting with cargo receptors such as SQSTM1/p62 and NBR1. These receptors promote cargo recruitment to LC3-II on the autophagosome surface, ensuring the efficient degradation of cellular materials [40] (Figure 1).

The final steps of the autophagy mechanism, fusion and degradation, begin with the transportation of autophagic vesicles to lysosomes for degradation. LC3 proteins attached to the autophagosome are recycled prior to fusion [41]. Fusion is facilitated by the coordinated action of SNARE proteins, such as STX17 and WAMP8, and lysosomal proteins, including LAMP2 and RAB proteins [42] (Figure 1). This process results in the formation of an autolysosome, where lysosomal proteases degrade the autophagic cargo. The breakdown products of autophagy, such as amino acids and fatty acids, are released into the cytosol and utilized as building blocks for various cellular processes.

## 4. Bi-Faceted Role of Autophagy in Cancer: Friend or Foe?

The role of autophagy in cancer development is complex and context-dependent, varying based on the type, stage, and genetic profile of the cancer [43]. In the early stages, autophagy acts as a protective mechanism by removing damaged organelles, misfolded proteins, and reactive oxygen species, thereby mitigating genomic instability and reducing the likelihood of cancer-causing mutations. However, in advanced stages, tumor cells exploit autophagy to adapt to stress and sustain their elevated metabolic demands, facilitating rapid growth and survival [44,45].

Impaired autophagy has been widely associated with tumorigenesis, genomic instability, and malignant transformation. Notably, research has shown that mice with only a single functional copy of the autophagy-related gene *Beclin-1* exhibit spontaneous tumor formation. Frequent allelic deletions of *Beclin-1*—ranging from 40% to 75%—have been identified in prostate, ovarian, and breast cancers, underscoring its role as a tumor suppressor gene [46,47]. *Beclin-1*, a key protein in the initiation of autophagy, interacts with and activates VPS34 via a highly conserved domain, driving autophagic activity. This functional domain, which activates VPS34, is likely integral to *Beclin-1*’s tumor-suppressive properties [48]. As a tumor suppressor, *Beclin-1*’s activity is regulated through interactions with proteins such as Bax-interacting factor-1 (Bif-1) and UVRAG, which enhance autophagy by stabilizing the *Beclin-1*–VPS34 complex [49]. Interestingly, several cancers—including stomach, bladder, prostate, breast, and colon cancers—exhibit disruptions in this pathway. These disruptions are frequently linked to monoallelic deletions or mutations in the *UVRAG* gene, along with reduced *Bif-1* expression [50].

The disruption of key members of the PI3K/Akt signaling pathway significantly impacts tumorigenesis and autophagy dysfunction [51,52]. Studies on HT-29 colon cancer cell lines (Cellosaurus ID: CVCL_0320) have demonstrated that the PTEN tumor suppressor promotes autophagy by inhibiting the Akt survival pathway through its phosphoinositide phosphatase activity [53]. Thus, loss-of-function mutations in the *PTEN* gene or constitutive activation of Akt lead to suppressed autophagic activity [53]. The inhibition of autophagy results in the accumulation of p62 aggregates, which contribute to DNA damage, oxidative stress, and cytotoxicity. Impaired autophagy has been consistently associated with poor prognostic outcomes in various cancers [54,55]. The absence of PTEN or aberrant PI3K/Akt signaling can reduce autophagic activity, further promoting tumorigenesis [53,54,55]. These findings highlight the importance of autophagy in controlling and regulating tumor initiation, with its dysregulation serving as a potential contributor to cancer progression.

In established tumors, autophagy plays a critical role in meeting the high metabolic demands of rapidly proliferating cancer cells. By breaking down macromolecules, autophagy protects tumor cells from metabolic stress-induced necrosis and recycles essential building blocks to sustain their elevated metabolism [56]. Furthermore, hypoxic conditions promote both HIF-1-dependent and HIF-1-independent autophagy, enhancing tumor cell survival under low-oxygen environments [57]. Given its importance in tumor progression, it is unsurprising that key autophagy-related genes can become genetically silenced or that pharmacological inhibition of autophagy can induce tumor cell apoptosis. For instance, in mouse models, deletion of the autophagy-related gene *FIP200* has been shown to significantly inhibit breast tumor growth [56].

Cancer cells harboring activating mutations in the *HRAS* or *KRAS* genes exhibit a pronounced dependence on autophagy, characterized by elevated basal autophagy rates even under normal growth conditions [54,58]. Studies on pancreatic cancer xenografts and murine models have demonstrated that inhibiting autophagy, either genetically or pharmacologically, leads to significant tumor regression [45,54,58]. In this context, autophagy serves as a critical mechanism for cancer cells to tolerate stress and secure alternative sources of nutrients and energy. By meeting their heightened metabolic demands, autophagy enables tumor cells to sustain growth and survival under challenging conditions.

## 5. Introduction to CML

CML is a malignant condition characterized by the abnormal proliferation of hematopoietic progenitor cells. It has been extensively studied and was the first malignancy to be associated with a chromosomal abnormality [58]. A hallmark of CML is the Philadelphia chromosome, resulting from a reciprocal translocation between chromosomes 9 and 22, designated t(9;22). This translocation creates the *BCR-ABL1* fusion gene, which is essential for diagnosing and monitoring the disease. The unrelenting activation of the BCR-ABL1 protein’s tyrosine kinase function is a key factor promoting disease pathogenesis. Abnormal kinase signaling induced by *BCR-ABL1* triggers a cascade of molecular events that disrupt normal cellular functions, leading to unchecked proliferation and an excessive accumulation of myeloid cells—a condition referred to as myeloid hyperplasia. This results in the indolent symptoms characteristic of the chronic phase of CML. If untreated, the disease can progress to blast crisis, a phase resembling acute leukemia. Common symptoms and signs of CML during the chronic phase include fatigue, splenomegaly, anemia, abdominal discomfort, and increased susceptibility to infections [59]. However, many patients may remain asymptomatic, with the condition often identified incidentally during routine medical evaluations [59].

The identification of the Philadelphia chromosome in individuals with CML was a landmark discovery, providing the first clear link between a chromosomal abnormality and a specific medical condition. The underlying biological mechanism of CML involves a genetic defect in blood stem cells responsible for producing myeloid cells. This defect arises from a chromosomal rearrangement, in which a segment of chromosome 9 fuses with chromosome 22, resulting in the Philadelphia chromosome and the formation of the distinctive *BCR-ABL* fusion gene.

**Figure 2:**
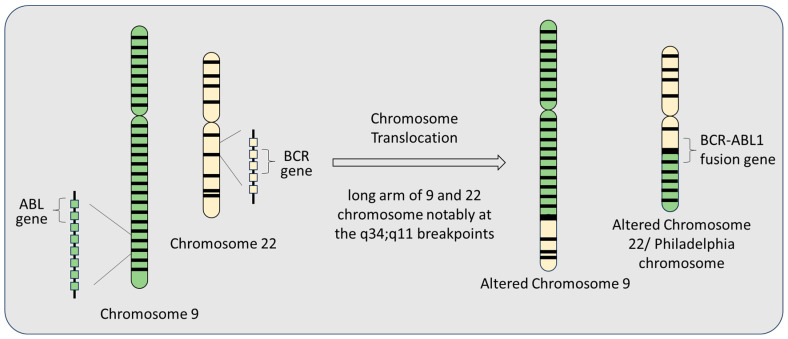
Occurrence of the Philadelphia chromosome results from a chromosomal translocation event where the genetic material is exchanged between chromosome 9 and chromosome 22.

A defining feature of CML is the Philadelphia chromosome, which arises from a specific chromosomal rearrangement known as a reciprocal translocation. This translocation occurs between the long (q) arms of chromosomes 9 and 22 at the precise breakpoints q34 and q11, respectively. It results in the juxtaposition of the *BCR* and *ABL1* genes, forming the *BCR-ABL* fusion gene. This fusion gene encodes a protein exhibiting aberrant tyrosine kinase activity, which is a critical driver of CML pathogenesis (Figure 2). The *BCR-ABL1* fusion gene combines the 3′ and 5′ ends of the *ABL1* gene (also referred to as *Abelson*) through a process of recombination. This typically involves the joining of intron 13 or 14 of the *BCR* gene with a 140-kilobase segment of the *ABL1* gene located between exons 1b and 2. However, the exact genomic breakpoints within the *BCR* and *ABL1* genes can vary significantly [60]. Despite this variability, the predominant outcome of mRNA splicing involves the creation of *BCR-ABL1* transcripts with either an e13a2 junction (joining *BCR* exon 13 and *ABL1* exon 2) or an e14a2 junction (joining *BCR* exon 14 and *ABL1* exon 2). These transcripts, formerly referred to as b2a2 and b3a2, respectively, encode the 210-kDa *BCR-ABL1* protein, which is instrumental in driving the progression of CML.

**Figure 3:**
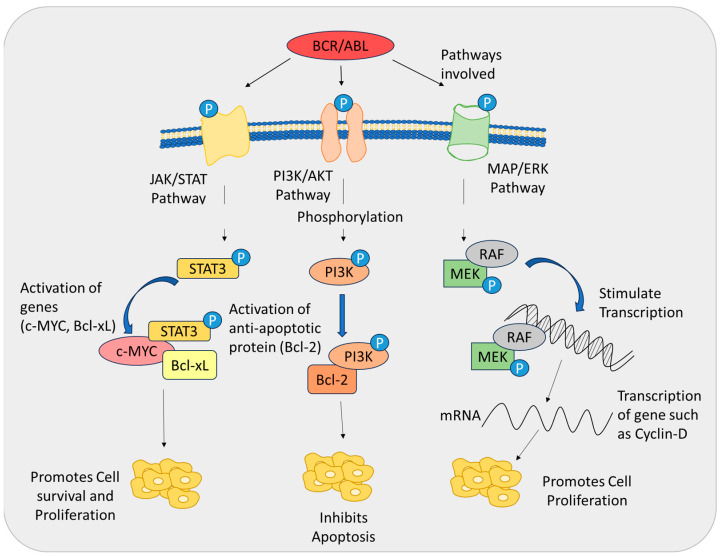
Activation of various signaling pathways by *BCR-ABL* protein, resulting in increased cell survival and reduced apoptosis.

The 210-kDa *BCR-ABL1* protein identified in CML contains over ten distinct functional domains derived from both the *BCR* and *ABL1* genes. Key domains from *BCR* include the Rho/GEF, Ser/Thr kinase, and dimerization (coiled-coil) domains. From *ABL1*, it incorporates the SH domains, actin- and DNA-binding domains, proline-rich region, nuclear localization signals, and nuclear entry signals. A critical feature of *BCR-ABL1* is its SH1 tyrosine kinase domain, which plays a central role in CML pathogenesis and has been a focal point of research. Additionally, tyrosine-177 within the Ser/Thr kinase domain is essential for the proper functionality of *BCR-ABL1* [61,62,63,64]. Interestingly, despite encompassing most of the *ABL1* gene, *BCR-ABL1* lacks the N-terminal myristoylation site present in the normal *ABL1* protein. In the native *ABL1* protein, myristoylation facilitates autoinhibition of *SH1* kinase activity by interacting with the myristoylation-binding pocket. The absence of this regulatory site in *BCR-ABL1* is thought to contribute to its constitutive kinase activity [65]. However, *BCR-ABL1* retains the myristoylation-binding pocket, enabling the development of pharmaceutical interventions aimed at targeting this region. These therapies have shown promise in reducing *BCR-ABL1* kinase activity allosterically, enhancing the effectiveness of treatments targeting CML [66]. *BCR-ABL1* drives CML pathogenesis by activating downstream signaling pathways that promote cellular proliferation, migration, and survival. It phosphorylates several substrates, including *CRKL*, *CBL*, and *STAT5*, which in turn activate diverse pathways such as *JAK/STAT*, *MAPK/ERK*, and *PI3K/Akt* (Figure 3).

The *JAK/STAT* signaling pathway plays a pivotal role in regulating cellular growth and development across various leukemias. This pathway is initiated by JAK receptors, which activate *STAT* transcription factors. In CML, the *BCR-ABL1* protein drives the activation of the *JAK/STAT* pathway, leading to the phosphorylation of *STAT5* [67]. Activated *STAT5*, in turn, regulates the expression of genes such as *c-Myc* and *Bcl-xL*, both of which are key contributors to enhanced cell survival and proliferation (Figure 3).

Studies using CML models have shown that *JAK2/STAT* activation is directly enhanced by *BCR-ABL1* kinase activity, promoting cell growth and survival [68,69]. Tyrosine-177, a critical site for BCR-ABL1 activation, has been identified as a phosphorylation target of *JAK2*. Mouse models in which *STAT5* was conditionally deleted both prior to and after the onset of *BCR-ABL1*-induced CML have demonstrated the indispensable role of *STAT5* in both the initiation and persistence of the disease [70].

*BCR-ABL1*-mediated phosphorylation of *PI3K* activates the *PI3K/Akt* signaling cascade, a critical pathway that promotes cell survival by inhibiting apoptosis. This pathway achieves anti-apoptotic effects primarily through the activation of proteins such as *Bcl-2*. *PI3K* proteins facilitate the communication between extracellular signals and transcription factor activation, thereby supporting cell growth and survival while suppressing programmed cell death [71]. *Akt*, a key downstream effector of *PI3K* signaling, plays a central role in this cascade. *BCR*-*ABL1* utilizes adaptor proteins such as Grb2/Gab2 [72] and CBL [73] to initiate *PI3K* signaling. Early studies revealed that *PI3K* activity is essential for hematopoietic cell transformation in the presence of *BCR-ABL1* [74]. Moreover, the *PI3K/Akt* pathway is crucial for sustaining CML, as inhibiting *PI3K* signaling effectively disrupts *BCR-ABL1*-driven oncogenic processes, leading to the elimination of early-stage CML cells [75]. In addition, *PI3K* activation stimulates the mTOR pathway [76], which regulates essential cellular processes such as protein synthesis, cell size, proliferation, and autophagy. Together, these interconnected pathways underscore the importance of *PI3K* signaling in the progression and maintenance of CML (Figure 3).

*BCR-ABL1* phosphorylates *RAF* and *MEK*, activating the *MAPK/ERK* signaling pathway, which drives cell proliferation. The *PI3K/Akt* cascade complements this by promoting the transcription of key genes involved in cell cycle progression, such as *cyclin D1*. The deregulation of these pathways is a major driver of cancer development, with the aberrant activation of Ras GTPases and *MEK* kinase serving as a critical mechanism [77]. Cellular differentiation is initiated through membrane receptor binding, which triggers the transcription of growth factor genes. *BCR-ABL1* amplifies Ras signaling by phosphorylating adaptor proteins such as Grb2 and Gab2, thereby enhancing cellular proliferation [78]. Notably, inhibiting Ras signaling has been shown to arrest the progression of *BCR-ABL1*-induced CML-like disease in mouse models [79]. Similarly, small-molecule *MEK* inhibitors have demonstrated efficacy in targeting early CML cells [80,81]. Despite progress in understanding these pathways, the specific contributions of the Ras-effector repertoire to disease progression remain poorly understood. One exception is *NF-κB*, a key transcription factor implicated in the progression of *BCR-ABL1*-induced CML. *NF-κB* is activated by *BCR-ABL1/Ras* signaling and plays a crucial role in sustaining the disease (Figure 3).

The advent of tyrosine kinase inhibitors (TKIs) revolutionized the management and treatment of CML, marking a significant therapeutic breakthrough. *TKIs* function by targeting the ATP-binding pocket of the *BCR-ABL* kinase, effectively inhibiting its activity. This disruption halts *BCR-ABL*-dependent signaling pathways that drive uncontrolled cell proliferation, ultimately inducing apoptosis in cancer cells. The success of *TKIs* in CML therapy underscores the pivotal role of *BCR-ABL* kinase activity in the molecular pathogenesis of the disease.

## 6. Deciphering the Role of Autophagy in CML

Chronic myeloid leukemia (CML) is characterized by the Philadelphia chromosome, resulting from a translocation between chromosomes 9 and 22, which produces the BCR-ABL fusion gene. This gene is a significant catalyst for clonal expansion in myeloid lineage cells and is linked to the existence of aberrant hematopoietic stem cells (HSCs) that possess this genetic modification. The introduction of tyrosine kinase inhibitors (TKIs) has revolutionized the treatment of chronic myeloid leukemia (CML) by selectively targeting the BCR-ABL protein, thereby obstructing its oncogenic function and addressing the unchecked cell growth characteristic of this leukemia [82,83]. Recent investigations have shown the mechanisms by which BCR-ABL regulates autophagy through multiple interrelated pathways, including those associated with endoplasmic reticulum (ER) stress. ER stress [84] occurs due to the buildup of misfolded or unfolded proteins in the endoplasmic reticulum, activating the unfolded protein response (UPR) to re-establish proteostasis. The UPR initiates multiple downstream signaling pathways, including *PERK-eIF2α*, *IRE1-XBP1*, and *ATF6*, which are associated with the induction of autophagy as a protective cellular strategy. In CML, extended ER stress promotes autophagy to eliminate misfolded proteins and mitigate cellular damage, hence improving cancer cell survival in unfavorable settings and fostering treatment resistance. A notable mechanism by which BCR-ABL affects autophagy is through the stimulation of the PI3K/AKT signaling pathway. This pathway is essential for facilitating cell survival and proliferation; BCR-ABL amplifies AKT activity, resulting in the overexpression of mTOR, a principal negative regulator of autophagy [85]. This activation leads to the mTOR-mediated inhibition of autophagic mechanisms, therefore promoting the survival of leukemic cells.

Furthermore, BCR-ABL directly engages with *Beclin-1*, a crucial regulator of autophagy. BCR-ABL, via phosphorylation of certain tyrosine residues, reduces *Beclin-1*’s capacity to assemble autophagic complexes with positive regulators like *UVRAG* and simultaneously increases its association with negative regulators like RUBICON, thereby further suppressing autophagy. In addition, BCR-ABL affects the production of *ATF5*, a transcription factor that enhances mTOR expression. Activation of the *PI3K/AKT* pathway by BCR-ABL enhances *ATF5* levels, resulting in increased mTOR activity and subsequent inhibition of autophagy. The *MAPK* signaling pathway is integral to this regulatory network; new discoveries indicate that BCR-ABL influences autophagy through interactions with LC3 family proteins via *MAPK* signaling [86]. The dual function of autophagy in chronic myeloid leukemia poses difficulties for treatment methods. Autophagy facilitates the survival of leukemic stem cells under stress circumstances, such as those caused by ER stress, hence contributing to resistance against TKIs. Conversely, the inhibition of autophagy has demonstrated the ability to sensitize CML cells to apoptosis, especially in scenarios when cells display resistance to TKIs. Notably, TKIs like imatinib can stimulate autophagy by suppressing mTORC1 activity and enhancing the expression of *Beclin-1* and ATG5. BCR-ABL utilizes many pathways—such as PI3K/AKT/mTOR signaling, modulation of *Beclin-1* and ATF5 regulation, *MAPK* signaling, and responses to ER stress—to carefully control autophagy in CML cells. Comprehending these systems underscores the intricacy of autophagic processes in CML and indicates prospective therapeutic targets that may improve treatment outcomes through the strategic modulation of these pathways.

Resistance to TKIs occurs in approximately 13% of CML patients [87], with autophagy playing a key role in this resistance. Both imatinib, a first-generation TKI, and ponatinib, a third-generation TKI, have been demonstrated to induce autophagy in resistant cells, which enhances both apoptosis and cytotoxic effects [88]. This autophagy provides cytoprotective benefits and reduces cell death, contributing to *TKI* resistance. Similarly, second-generation *TKIs*, such as nilotinib and dasatinib, also induce autophagy, apoptosis, and cytotoxicity in resistant cells [83]. Efforts to overcome TKI resistance have focused on suppressing autophagy to enhance *TKI* efficacy. A notable advancement involves improving the effectiveness of ponatinib by knocking down ATG7 or using hydroxychloroquine (HCQ) to inhibit mTOR and disrupt autophagic processes [89]. However, HCQ, the only FDA-approved autophagy inhibitor, has shown inconsistent efficacy in fully inhibiting autophagy. As an alternative, second-generation inhibitors such as *Lys05* and *PIK*-*III* are under investigation. *PIK-III* specifically targets *VPS34*, while *Lys05* acts as a lysosomotropic agent. Both inhibitors have been shown to suppress autophagy and promote cell death, leading to a reduction in initial CML cell populations [90]. Autophagy has also been implicated in resistance to other anticancer agents, including 20(S)-ginsenoside Rh2 [91] and diosgenin. These compounds induce autophagy alongside drug-mediated cell death in CML cells, which may hinder apoptosis and contribute to the development of drug resistance, particularly in K562 and U937 cells [92].

One study by Mitchell et al. [89] demonstrated that ponatinib-resistant CML cells can develop BCR-ABL-independent resistance through alternative activation of the mTOR pathway. The inhibition of mTOR in these cells not only sensitized them to apoptosis but also induced compensatory autophagy. This study emphasized combining mTOR inhibitors like NVP-BEZ235 with autophagy inhibitors. Hydroxychloroquine (HCQ) significantly enhanced the apoptosis of ponatinib-resistant CML cells both in vitro and in vivo, suggesting a potential therapeutic avenue to address resistance.

Another investigation by Elshazly et al. [93] examined the elevated levels of autophagy in resistant CML cell lines compared to their parental counterparts in which lapatinib-resistant cells exhibited increased autophagosome accumulation. When autophagy inhibitors were combined with lapatinib, there was a marked increase in cell death and a reduction in colony formation. This underscores the protective role of autophagy in mediating resistance and highlights how its inhibition can enhance the efficacy of TKIs.

Additionally, a study by Li et al. [94] explains the targeting energy metabolism, a strategy to overcome TKI resistance. Research demonstrated that combining 2-deoxy-D-glucose (2-DG), a glycolysis inhibitor, with imatinib resulted in significant reductions in cellular ATP levels and induced autophagic cell death. This effect was particularly pronounced in CML cells harboring the T315I mutation, one of the most challenging mutations conferring TKI resistance. These findings highlight that metabolic modulation, alongside autophagy inhibition, can sensitize resistant CML cells to TKIs.

In 2010, Altman and colleagues [95] developed a conditional model for hematopoietic cell culture by employing Cre-mediated excision of ATG3 to disrupt autophagy in cells reliant on growth stimuli. Their findings revealed that basal autophagy, though present at low levels, was highly dependent on autophagic processes in cells expressing *BCR-ABL*. Inhibiting autophagy through ATG3 deletion led to significant effects, including increased phosphorylation and the accumulation of *p53*, as well as the upregulation of *p53* target genes, such as *p21* and the pro-apoptotic *Bcl-2* family protein *Puma*. Additionally, researchers observed elevated levels of *ƴH2AX*, a marker of DNA double-strand breaks, following ATG3 deletion. However, it remained unclear whether autophagy inhibition affected mitochondrial number or function. This uncertainty is notable, as mitochondrial activity could potentially contribute to increased DNA damage, a phenomenon previously reported in healthy hematopoietic stem cells (HSCs) [96,97].

Sheng and colleagues [85] demonstrated that *FOXO4* plays a crucial role in the *PI3K/Akt* signaling pathway, through which *BCR-ABL* transcriptionally activates activating transcription factor 5 (*ATF5*). *ATF5*, in turn, stimulates *mTORC1* transcription, which is required to suppress autophagy. These findings indicate that *BCR-ABL* enhances not only *mTORC1* kinase activity but also its transcriptional expression. Furthermore, the study revealed that the initiation of autophagy by imatinib depended on the suppression of the *PI3K/Akt/mTORC1* pathway. The constitutive activation of *PI3K* was shown to impair imatinib’s ability to induce autophagy, highlighting the interplay between these pathways. In a separate investigation, Yu et al. [98] reported that imatinib treatment reduced the expression of microRNA-30a (miR-30a) in CML cells. This reduction led to autophagy activation, as evidenced by increased expression of *Beclin-1* and ATG5, key regulators of autophagy.

These findings collectively indicate that *BCR-ABL* suppresses autophagy through two distinct mechanisms. The first mechanism is mTORC1-independent and involves the induction of *mir-30a* expression, while the second is mTORC1-dependent, relying on increased mTORC1 activity and expression. The inhibition of mTOR using OSI-027, an inhibitor targeting both mTORC1 and mTORC2 complexes, was shown to induce protective autophagy in K562 cells. This suggests that active mTORC1 primarily mediates the suppression of autophagy. Furthermore, combining OSI-027 with CQ-mediated autophagy inhibition significantly enhanced apoptosis compared to OSI-027 treatment alone [99]. These observations indicate that autophagy may be critical for promoting survival and reducing apoptotic responses in CML cells following mTOR inhibition. Therefore, combining potent mTOR inhibitors with autophagy inhibitors could represent an effective therapeutic strategy to maximize treatment efficacy. Interestingly, the role of autophagy in CML appears to be complex. While autophagy typically functions as a tumor suppressor in healthy hematopoietic stem cells (HSCs), its role in CML differs, suggesting that targeting autophagy in this context holds potential as a therapeutic approach.

Autophagy plays a vital role in maintaining cellular homeostasis and is intricately regulated by the aforementioned key pathways, mTOR, PI3K/Akt, and AMPK, which are also central to CML pathogenesis. Interestingly, these pathways are also implicated in other diseases, such as neurodegenerative [100] and metabolic disorders [45]. In a study focused on neurodegenerative diseases [100], metformin was shown to induce autophagy through the AMPK-mTOR-ULK1 pathway and lysosomal-dependent chaperone-mediated autophagy via TAK1-IKK α/β-Hsc70 signaling, which reverses the molecular and behavioral phenotypes of Alzheimer’s disease. Similarly, resveratrol activates autophagy by inhibiting the Akt/mTOR pathway, improving cognitive function, and enhancing mitophagy through increased acidic vesicular organelle formation, LC3-II/LC3-I ratio, and expression of Parkin and *Beclin-1*. In metabolic disorders, such as atherosclerosis [101], the PI3K/Akt and mTOR pathways are also pivotal in regulating autophagy. For example, high glucose concentrations suppress the PI3K/Akt pathway, leading to proliferative dysfunction in endothelial cells (ECs), while hyperinsulinemia activates the *MAPK* pathway, promoting endothelial dysfunction and proatherosclerotic events. Caveolin-1, a marker for caveolar organelles, regulates autophagy by interacting with LC3 and the ATG5/ATG12/ATG16 complex during autophagosome formation, facilitating caveolin-1 degradation. In CML, autophagy supports leukemic stem cell survival and TKI resistance by leveraging the same mTOR, PI3K/Akt, and AMPK pathways, which are also critical in the pathogenesis of neurodegenerative and metabolic disorders.

## 7. Targeting Autophagy as a Therapeutic Intervention for CML

Currently, there are no clinically approved autophagy inhibitors specifically developed for therapeutic use. However, the anti-malarial drug HCQ, approved for treating inflammatory conditions such as rheumatoid arthritis, has demonstrated the ability to suppress autophagy [102]. HCQ accumulates in lysosomes, where it raises pH levels and disrupts the degradation of autophagic proteins. It has been widely studied as an autophagy inhibitor in over 40 clinical trials involving cancer patients, either as a standalone treatment or in combination with cytotoxic therapies [103,104]. Autophagy has been implicated in the persistence of CML stem cells, offering both a challenge and an opportunity for therapeutic intervention. Carew and colleagues [105] demonstrated that chloroquine (CQ), an autophagy inhibitor, enhanced the efficacy of the HDAC inhibitor suberoylanilide hydroxamic acid (SAHA) in primary CML cells resistant to imatinib. These findings suggest that autophagy contributes to the survival and drug resistance of *BCR-ABL*-driven CML cells. Recent studies have also identified alternative autophagy inhibitors, including macrolide antibiotics such as azithromycin [106] and clarithromycin [107], as well as desmethylclomipramine, a metabolite of a drug used for psychiatric conditions [107]. Carella et al. [108] observed improved treatment outcomes in four patients with advanced CML resistant to *TKIs* when clarithromycin was added to their therapy. However, further research is needed to determine whether the observed benefits are directly related to autophagy inhibition. Carella’s lab is currently investigating the autophagy-inhibiting potential of the clarithromycin concentrations used in their study.

Additionally, other potential autophagy inhibitors, including Lys05 [109], mefloquine [110], and spautin-1 [111], have shown promising results in preclinical studies. Among the autophagy inhibitors evaluated beyond HCQ, Lys05, a trihydrochloride salt of the lead compound Lys01 has emerged as a promising candidate due to its increased potency and solubility for in vivo investigations. McAfee et al. [109] demonstrated that Lys01 was shown to be a 10-fold more potent autophagy inhibitor than HCQ, with its water-soluble derivative, Lys05, exhibiting enhanced lysosomal accumulation and deacidification. This pathway leads to significantly impaired autophagy and subsequent suppression of tumor growth. The Lys05 treatment resulted in a significant dose-dependent elevation of the LC3II/LC3I ratio, indicating increased autophagy inhibition, and also resulted in the accumulation of the autophagy cargo protein p62. Furthermore, following 14 days of treatment, Lys05-treated tumors had a sixfold increase in autophagic vesicles (AVs) relative to control-treated tumors, accompanied by significant tumor necrosis. These findings underscore Lys05’s effectiveness in preclinical cancer models and its promise as an autophagy inhibitor for therapeutic use in CML. Although research directly evaluating Lys05 in CML is scarce, its significant efficacy against autophagy-related survival mechanisms in alternative tumor models highlights its potential as a candidate for addressing TKI resistance in CML.

In addition to HCQ, celecoxib, a cyclooxygenase-2 (COX-2) inhibitor, has demonstrated potential anti-tumor benefits in solid tumors and has recently been examined for its anti-CML properties. Although the precise mechanisms of its activity in CML remain unclear, current research by Lu et al. [112] highlights its function as an autophagy inhibitor. In contrast to other inhibitors targeting the mTOR route to modulate autophagy, celecoxib does not influence phosphorylated mTOR or 4EBP proteins, signifying that it suppresses autophagy independently of the mTOR system. It was found to block autophagic flux at its late stages, leading to the accumulation of autophagic vesicles, similar to the effect of chloroquine (CQ) in inhibiting autophagy. These data indicate that celecoxib impedes the disruption of autophagic vesicles, resulting in the buildup of autophagy cargo. The research highlights celecoxib’s potential as a dual-function agent that suppresses COX-2 and targets autophagy, positioning it as a possible contender for overcoming TKI resistance in CML.

Apart from the use of autophagy inhibitors, it is well established that the activation of the ULK1 complex initiates autophagy. This makes ULK1, a druggable serine/threonine kinase, a promising target for autophagy suppression [113,114,115]. In a study on primitive leukemia cells, Ianniciello et al. [116] explored the role of ULK1 in regulating autophagy, differentiation, central carbon metabolism, and drug sensitivity. Their findings revealed that treatment with *TKIs* activated *AMPK* and *ULK1*, proteins integral to autophagy initiation. This activation led to increased autophagy in both patient-derived CML cells and cell lines with inherently low autophagy levels. Significantly, the researchers demonstrated that TKI-induced autophagy could be effectively reduced by either genetically deleting *ULK1* or administering MRT403, a specific *ULK1* kinase inhibitor. This intervention shifted the energy metabolism of CML cells from glycolysis to mitochondrial respiration, resulting in enhanced differentiation and oxidative stress mediated by reactive oxygen species (ROS). Moreover, the dual inhibition of *BCR-ABL* and *ULK1* in animal models restored the normal ratio of immature myeloid and erythroid cells in the bone marrow and effectively targeted therapy-resistant CML leukemic stem cells. These findings support the idea that selective autophagy inhibition can enhance anticancer therapy. MRT403 is the first *ULK1*-specific inhibitor approved for preclinical animal studies in cancer and other diseases [117]. Additionally, the experimental ULK1 inhibitor DCC-3116 has entered Phase 1 clinical trials to evaluate its potential in treating RAS-driven cancers, both as a monotherapy and in combination with *MAPK* inhibitors. This ongoing trial, registered under ClinicalTrials.gov, identifier NCT04892017, aims to assess the therapeutic potential of *ULK1* inhibition as a novel approach for certain tumor types [117].

The concurrent targeting of autophagy and BCR-ABL inhibition has surfaced as a viable approach to address the limitations associated with tyrosine kinase inhibitors (TKIs) in chronic myeloid leukemia (CML). Although TKIs like imatinib, dasatinib, and nilotinib have transformed CML therapy, their effectiveness is frequently diminished by resistance mechanisms, including autophagy-mediated survival pathways. Strategies using dual inhibition, such as the combination of BCR-ABL inhibitors with autophagy inhibitors, demonstrate promise in addressing these issues by concurrently targeting numerous survival processes.

To enhance treatment outcomes in CML, clinical trials have investigated the combination of TKIs with additional therapeutics to facilitate TKI-induced apoptosis and reduce resistance mechanisms. Research combining ruxolitinib with TKIs has demonstrated promising molecular responses; however, overall survival and progression-free survival have not improved (NCT03610971, Phase 2) [118]. The investigation of asciminib in conjunction with TKIs has been conducted (NCT04838041, Phase 2) [119], with smaller trials evaluating asciminib as a monotherapy or in combination with nilotinib, imatinib, or dasatinib (NCT04877522, Phase 4) [120]. Further trials have examined KRT-232, an innovative oral small molecule inhibitor of MDM2, alongside TKIs (NCT04835584, Phase 1/2) [121] and the novel drug ABL001 in conjunction with dasatinib, prednisone, and blinatumomab (NCT03595917, Phase 1) [122].

ASTX727 and dasatinib have been utilized in conjunction for the treatment of newly diagnosed Philadelphia chromosome or BCR-ABL-positive CML [123]. Moreover, hydroxychloroquine (HCQ) [89], a recognized autophagy inhibitor, has surfaced as a significant possibility for combination therapy by addressing autophagy-mediated survival pathways that facilitate TKI resistance. Strategies of dual inhibition utilizing TKIs and autophagy inhibitors not only undermine compensatory mechanisms but also provide a more precise method for eradicating leukemic stem cells (LSCs). This differs from conventional combinations of chemotherapy or immunomodulators, which frequently lead to increased systemic toxicity or neglect the significance of autophagy in resistance. Clinical trials with HCQ have yielded inconsistent outcomes due to difficulties in fully inhibiting autophagy; nonetheless, the creation of more effective inhibitors offers the potential for enhancing dual inhibition as a viable alternative to existing treatments.

Recent research by Iralde-Lorente et al. [124] has identified phosphate-containing compounds as potential inhibitors of the 14-3-3/c-Abl protein–protein interaction (PPI), a pathway implicated in the pathogenesis of chronic myeloid leukemia (CML). Pyridoxal phosphate (PLP) and inosine monophosphate (IMP) have been identified as binding agents to human 14-3-3σ, specifically targeting the protein’s amphipathic groove and functioning as weak inhibitors of the 14-3-3/c-Abl protein–protein interaction (PPI). The findings were corroborated by techniques including NMR, SPR, and fluorescence polarisation assays. These compounds demonstrated minimal cytotoxicity in human Hs27 fibroblasts, suggesting selectivity for cancer cells. This selectivity highlights the potential of targeting 14-3-3σ interactions as a therapeutic approach in c-Abl-dependent cancers, such as CML. The identification of these inhibitors facilitates the development of drugs targeting specific protein–protein interactions involved in oncogenic signaling pathways.

Chronic myeloid leukemia (CML) has focused on refining diagnostic approaches and exploring innovative therapeutic strategies to overcome resistance mechanisms and improve patient outcomes. These include RNA-based targeted gene sequencing, CAR-T cell therapy, and proteolysis-targeting chimeras (PROTACs), all of which offer new avenues to address the limitations of current treatments.

Traditional diagnostic methods, such as RNA-based targeted gene sequencing [125], primarily depend on DNA sequencing and exhibit limitations in identifying mutations that affect RNA splicing or expression levels, which may result in the oversight of clinically significant alterations. RNA-based targeted gene sequencing has emerged as a more effective diagnostic tool, providing enhanced sensitivity and specificity in the identification of mutations linked to CML. This method enables the detection of rare mutations, alternative splicing events, or gene fusions through direct analysis of transcripts, which may be missed by DNA-based approaches. The enhancement of diagnostic precision is essential for customizing treatment approaches, especially in detecting mutations that lead to resistance against tyrosine kinase inhibitors (TKIs). RNA sequencing can detect BCR-ABL mutations, including the T315I mutation, which can lead to the ineffectiveness of specific TKIs. The early identification of these mutations facilitates a more accurate selection of alternative therapies, including third-generation TKIs such as ponatinib or experimental agents that target novel resistance pathways. This technique facilitates a comprehensive understanding of disease biology by revealing mutations associated with disease progression and relapse.

Chimeric antigen receptor T-cell (CAR-T) therapy [126] has revolutionized the treatment landscape for hematologic malignancies, and its potential use in CML is currently under investigation. In pediatric patients with chronic myeloid leukemia experiencing recurrent relapses into the lymphoid blast phase, CD19-directed CAR-T cells have shown significant efficacy under compassionate use protocols. Patients who frequently do not respond to standard therapies because of resistance or intolerance demonstrated sustained remission following CAR-T therapy, underscoring its therapeutic potential. Kramp et al. [126] demonstrated that CAR-T therapy was well tolerated, exhibiting minimal adverse effects, while successfully achieving complete B-cell aplasia and sustaining detectable CAR-T cells in peripheral blood for up to 12 months. This sustained remission highlights the efficacy of CAR-T cells in eliminating resistant leukemic cells that escape standard therapies. This approach is in experimental stages for CML but its success in pediatric cases establishes a basis for further research and clinical trials. CAR-T therapy may demonstrate enhanced efficacy when combined with other treatments, such as TKIs, to address various mechanisms of resistance.

Proteolysis-targeting chimeras (PROTACs) [127] represent an emerging therapeutic strategy aimed at degrading specific oncogenic proteins through the ubiquitin–proteasome system. In contrast to traditional inhibitors that solely obstruct the function of target proteins, PROTACs promote their degradation, resulting in a more sustained therapeutic impact. In chronic myeloid leukemia (CML), PROTACs that target epigenetic regulators, including histone deacetylases (HDACs) and bromodomain and extraterminal domain (BET) proteins, have demonstrated considerable potential in preclinical models. Epigenetic dysregulation is integral to the pathogenesis of chronic myeloid leukemia (CML), facilitating the survival of leukemic stem cells and their resistance to tyrosine kinase inhibitors (TKIs). The selective degradation of epigenetic modulators by PROTACs disrupts the transcriptional programs sustaining CML cells, providing a novel mechanism to address resistance. Furthermore, PROTACs can be engineered to target various resistance-associated proteins, including mutant variants of BCR-ABL, offering an alternative to next-generation TKIs. Preclinical studies indicate that PROTACs can synergize with TKIs, thereby enhancing efficacy and reducing the development of resistance.

Several unresolved questions remain regarding the therapeutic application of autophagy inhibition. One of the main challenges is the development of more effective and targeted autophagy inhibitors suitable for clinical use. Addressing this issue will require close collaboration between the pharmaceutical industry and academic researchers. Additionally, it is essential to test these potential inhibitors using appropriate in vitro and animal models to ensure their safety and efficacy. It is important to recognize that drugs targeting different stages of autophagy may produce varying effects. For instance, while targeting the initiation or formation of autophagosomes may prove beneficial, suppressing the later stages of autophagy could be detrimental to cellular health. This underscores the need for a nuanced understanding of how different autophagy pathways impact cancer and other diseases. Another significant challenge lies in effectively monitoring autophagy in patient samples. Given the dynamic nature of this process, there is a critical need to develop minimally invasive, standardized, and reliable methods to measure autophagy consistently. Accurate and reproducible monitoring is essential for assessing the therapeutic impact of autophagy inhibitors in clinical contexts. Ongoing clinical trials investigating the use of HCQ to inhibit autophagy in cancer patients may provide valuable insights into some of these challenges. These studies hold promise for determining the feasibility of autophagy inhibition as a strategy to enhance the efficacy of existing anticancer therapies, paving the way for future advancements in this field.

The list of the drugs under clinical trial for the treatment of CML that are *BCR/ABL*-positive at Phase 3 or Phase 4 is mentioned in Table 1.

## 8. Concluding Remarks and Future Perspectives

Autophagy, a conserved cellular process across various organisms, is essential for maintaining cellular homeostasis by eliminating damaged organelles and protein aggregates. This process is regulated by a complex signaling network involving numerous proteins and molecules. The dysregulation of autophagy has been implicated in a broad spectrum of diseases, including cancer, where its impact on tumor growth can be either promoting or inhibitory depending on the specific circumstances. CML is driven by the presence of a unique abnormal protein, *BCR-ABL1*, which results from a genetic fusion event. This misfolded protein triggers the transformation of hematopoietic progenitor cells into malignant cancer cells. Notably, autophagy is important for the survival of CML stem cells and contributes to resistance against *TKIs*, which are the current standard of care for CML. These insights highlight the dual role of autophagy in CML pathogenesis and its potential as a therapeutic target. Numerous clinical studies have explored the potential of autophagy inhibition to enhance the effectiveness of *TKI* therapy in treating CML. These studies have primarily focused on chloroquine and hydroxychloroquine, which have demonstrated the ability to inhibit autophagy and improve the anti-cancer efficacy of *TKIs* in preclinical models. Additional potential autophagy inhibitors, including azithromycin, clarithromycin, desmethylclomipramine, Lys05, mefloquine, and spautin-1, have also shown promise in preclinical research.

While these studies provide preliminary evidence supporting the benefits of autophagy inhibition in CML, significant challenges and unanswered questions remain. These include the need for more potent and selective autophagy inhibitors, the potential toxicity associated with blocking later stages of autophagy, and the development of reliable and minimally invasive methods for monitoring autophagy in patient samples. Research into the role of autophagy in CML and its potential as a therapeutic target is ongoing. Further studies are needed to determine the most effective strategies for autophagy inhibition and to fully evaluate the risks and benefits of this approach. A comprehensive understanding of these factors will be critical for optimizing the therapeutic potential of targeting autophagy in CML.

## Figures and Tables

**Table 1:** Ongoing therapeutic clinical trials for the treatment of CML.

Clinical Trial No.	Phase	Targeted Drug	Title of Study	References
NCT03610971	Phase 2	Ruxolitinib BCR-ABL Tyrosine Kinase Inhibitor (TKI)	Treatment Free Remission After Combination Therapy With Ruxolitinib Plus Tyrosine Kinase Inhibitors	[118]
NCT04838041	Phase 2	Asciminib 40 MG Asciminib 40 MG Asciminib 80 MG Imatinib Nilotinib Dasatinib	Protocol Number: HJKC3-0003. Treatment Free Remission After Combination Therapy With Asciminib (ABL001) Plus Tyrosine Kinase Inhibitors (TKI) in Chronic Phase Chronic Myeloid Leukemia (CP-CML) Patients Who Relapsed After a Prior Attempt at TKI Discontinuation	[119]
NCT04877522	Phase 4	Asciminib Imatinib Nilotinib Bosutinib Dasatinib	Asciminib Roll-over Study	[120]
NCT04835584	Phase 1, Phase 2	KRT-232 Dasatinib Nilotinib	KRT-232 and TKI Study in Chronic Myeloid Leukemia	[121]
NCT03595917	Phase 1	ABL001 Dasatinib Prednisone Blinatumomab	ABL001 + Dasatinib + Prednisone + Blinatumomab in BCR-ABL+ B-ALL or CML	[122]
NCT02602314	Phase 4	Imatinib Nilotinib	Sustained Treatment-free Remission in *BCR-ABL*+ Chronic Myeloid Leukemia (SUSTRENIM)	[128]
NCT03722420	Phase 3	Radotinib Imatinib	Randomized Evaluation of Radotinib Versus Imatinib in Phase III Study for Efficacy with Chinese Patients	[129]
NCT04666259	Phase 3	ABL001	Asciminib in Monotherapy for Chronic Myeloid Leukemia in Chronic Phase (CML-CP) With and Without T315I Mutation (AIM4CML)	[130]
NCT03106779	Phase 3	Asciminib Bosutinib	Study of Efficacy of CML-CP Patients Treated with ABL001 Versus Bosutinib, Previously Treated With 2 or More TKIs	[131]
NCT02890784	Phase 3	Dasatinib	Dasatinib Holiday for Improved Tolerability (DasaHIT)	[132]
NCT04971226	Phase 3	Imatinib Nilotinib Bosutinib Dasatinib Asciminib	A Study of Oral Asciminib Versus Other *TKIs* in Adult Patients with Newly Diagnosed Ph+ CML-CP	[133]
